# Dealloyed porous gold anchored by *in situ* generated graphene sheets as high activity catalyst for methanol electro-oxidation reaction†

**DOI:** 10.1039/c9ra09821f

**Published:** 2020-01-09

**Authors:** Hui Xu, Shuai Liu, Xiaoliang Pu, Kechang Shen, Laichang Zhang, Xiaoguang Wang, Jingyu Qin, Weimin Wang

**Affiliations:** State Key Laboratory for Modification of Chemical Fibers and Polymer Materials, International Joint Laboratory for Advanced Fiber and Low-dimension Materials, College of Materials Science and Engineering, Donghua University Shanghai 201620 China xuhui199278@dhu.edu.cn; Key Laboratory for Liquid-Solid Structural Evolution and Processing of Materials, Ministry of Education, Shandong University Jinan 250061 China weiminw@sdu.edu.cn; Ulsan Ship and Ocean College, Ludong University Yantai 264025 China; School of Engineering, Edith Cowan University 270 Joondalup Drive, Joondalup Perth WA6027 Australia; Laboratory of Adv. Mater. & Energy Electrochemistry, Taiyuan University of Technology Taiyuan 030024 China

## Abstract

A novel one-step method to prepare the nanocomposites of reduced graphene oxide (RGO)/nanoporous gold (NPG) is realized by chemically dealloying an Al_2_Au precursor. The RGO nanosheets anchored on the surface of NPG have a cicada wing like shape and act as both conductive agent and buffer layer to improve the catalytic ability of NPG for methanol electro-oxidation reaction (MOR). This improvement can also be ascribed to the microstructure change of NPG in dealloying with RGO. This work inspires a facile and economic method to prepare the NPG based catalyst for MOR.

## Abstract 2

A novel one-step method to prepare the nanocomposites of reduced graphene oxide (RGO)/nanoporous gold (NPG) is realized by chemically dealloying an Al_2_Au precursor.

## Graphical abstract

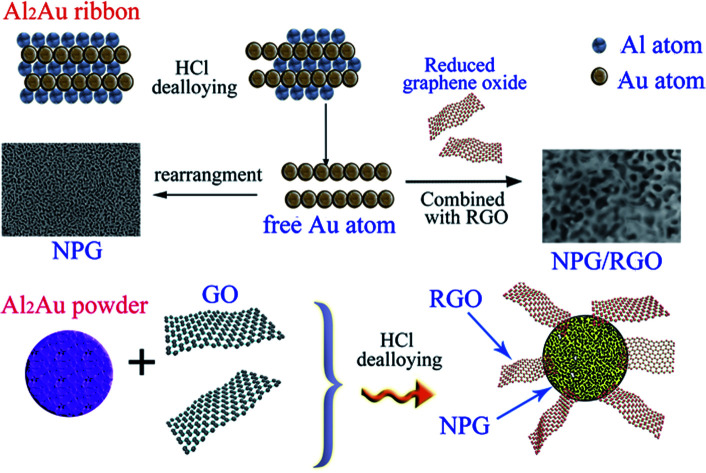

## 1. Introduction

Traditionally gold has not been considered as a useful catalyst due to its chemical inertness. However, recent studies have found that when the feature size decreases to the nanometer scale, the nanostructured gold can exhibit unusual catalytic properties in several important reactions such as energy generation, environment protection and biosensing.^1–6^ Much effort has been devoted to develop novel nanostructured gold with various morphologies to exploit the innovative nanoscale chemical effects.^7,8^ Nanoporous gold (NPG) with high surface area can be fabricated by selectively etching an alloy, *i.e.*, dealloying, and the as-evolved bi-continuous open ligament/channel structure enables fast transport of medium molecules and electrons, which is particularly beneficial for electrocatalysis.^9–11^ Over the past decade, methanol electro-oxidation reaction (MOR) has been receiving great attentions due to its importance in direct methanol fuel cells (DMFCs).^12–14^ Generally, the surface of Pt or Pt based catalysts is easy to be poisoned by the intermediate species formed in MOR, which further declines the catalytic activity.^15,16^ Surprisingly, the unique oxidation mechanism of MOR on NPG electrode protects it free from poisoning.^12,17–20^ And the specific reaction steps are described in discussion parts.

The excellent catalytic performance of NPG mainly depends on the uniform and small nanopore size.^21^ Several reports have proved that by adjusting the precursor alloy component,^22–24^ dealloying time,^25^ dealloying temperatures,^26^ and etching solutions,^27–29^ the pore size of nanoporous gold can be tuned. Bäumer *et al.*^30^ have found that surface Ag impurities are crucial for the remarkable oxidative activity of NPG. Our previous report noted that NPG with a three-dimensional (3D) bicontinuous interpenetrating ligament-channel structure can be prepared by dealloying the melt spun Al_2_Au ribbon precursors with different circumferential speeds (*S*_c_).^31^ We also can further enhance the catalytic activity for MOR by adjusting magnetic field in dealloying.^32^

Recently, given its large specific surface area,^33^ unique electronic properties,^34–37^ and excellent mechanical strength and flexibility,^38–40^ graphene,^41^ a flat monolayer of carbon atoms densely packed into a 2D honeycomb lattice, is a promising support to disperse catalytically active metal nanoparticles.^42–44^ Several monometallic nanoparticles, such as Pd,^45^ Pt,^46^ Au,^47^ and Ag^48^ have been successfully dispersed on graphene, and the hybrids exhibit an impressive catalytic performance in various reactions. A strong metal–graphene interaction was revealed and may contribute to the enhanced catalytic performances of the supported monometallic nanoparticles.^42,49^ In the aspect of LIB application, the silicon/reduced graphene oxide (Si/RGO) nanocomposites show a high reversible capacity and excellent rate capability.^50^ The RGO anchoring could act as both conductive agent and buffer layer for Si volume change in the charge–discharge process. According to the Feng *et al.*,^51–53^ by employing graphene as outer shell to completely encapsulate metals, it can effectively avoid the inside metals from being destroyed in harsh environments. And we are devoted to research the effect of graphene incorporating Au on the pore size and catalytic activity of NPG.

**Fig. 1:**
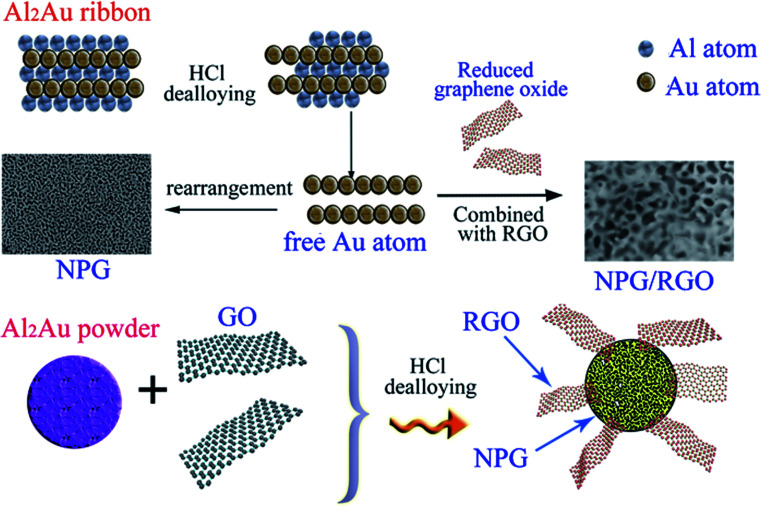
Schematic illustration of the formation of nanocomposite ribbons and powders.

Herein, we provide a facile method to fabricate nanocomposites of NPG and RGO by dealloying Al_2_Au ribbons and powders in the acid environment containing certain graphene oxide solution. During the dealloying process, GO was reduced by the reaction between Al atoms and HCl solution, further forming an Al–Au/RGO structure; then NPG/RGO nanocomposites were fabricated accompanying with the removal the residual Al in the HCl solution (Fig. 1). In a GO solution, the surface of the Al–Au alloy is positively charged accompanied by the Al atoms loses electrons, then the GO sheets can be easily partially reduced after accepting electrons because the abundant oxygen-containing functional groups exist in the surface of GO. The partially reduced GO sheets become hydrophobic and easy to restack on the Al–Au alloy surface in order to minimize their surface energy. The residual Al can be removed by adding excessive HCl solution, forming nanoporous gold/reduced graphene oxides nanocomposites. In this process, thanks to their flexibility and ductility, RGO sheets intersperse randomly on the sphere-structure of NPG, further forming an armour-like structure.

## 2. Experimental section

### Materials synthesis

A graphene oxide (GO) aqueous solution was synthesized by the typical modified Hummers' method.^54^ The pure bulk metals (Au, 99.99%; Al, 99.99%) were purchased from Zhaojin Group Co., Ltd (Yantai, China), and Trillion Metals Co., Ltd (Beijing, China), respectively. Concentrated hydrochloric acid (AR, 37.5 wt%), concentrated sulfuric acid (AR, 98.3 wt%), potassium hydroxide (99.99 wt%), methanol (GR) *etc.* were supplied by Sinopharm Chemical Reagent Co., Ltd. Firstly, the influence of graphene oxide (GO) on the dealloying process of precursor Al_2_Au ribbons was investigated. The prepared details can be referenced from our former works.^31^ Typically, several Al_2_Au ribbons (totally about 3 g) were firstly soaked in certain GO-concentrated solution, then 10 wt% HCl solution was slowly added into graphene oxide solutions, the final GO concentration *C*_GO_ of which varies from 0 to 100 ppm, respectively. Until no further bubbles were observed, the etching process finished. The nanocomposite ribbon samples dealloyed in the solutions with *C*_GO_ = 0, 50, and 100 ppm were labeled as S0, S1 and S2, respectively.

Subsequently, according to the consequences of the NPG/RGO ribbons, the effect of GO on the dealloying process of Al_2_Au powders was further investigated. The Al_2_Au ingot was ball-milled into micro order powders using the high energy ball-milling. 1 g Al_2_Au powders were first dispersed in GO-concentrated solution. After adding HCl solution, the final *C*_GO_ of dealloying solution is 50 ppm. The as-prepared nanocomposites powders were obtained after a series of centrifugation and washing. For the synthesis of blank NPG, the dealloying process is similar and the dealloying solution does not contain graphene oxide. Hereafter, the obtained NPG and NPG/RGO powder samples were marked as P0 and P1, respectively.

### Microstructure characterization

X-ray diffraction (Rigaku D/MAX 2500/PC diffractometer) was performed with Cu Kα radiation. The lattice constant *a*_0_ and the preferred orientation factors *F* of (111) faces of samples were calculated by the extended Bragg equation and Lotgering method (eqn (S1) and (S2)†).^55,56^ The specific calculation methods were displayed in ESI.† The microstructure morphologies of nanocomposite ribbons and powders were investigated by a scanning electron microscope (SEM, Zeiss SUPRA 55) and a transmission electron microscope (TEM, JEOL JEM-2100) with selected-area electron diffraction (SAED) patterns. Some TEM specimens were also observed using high-resolution TEM (HRTEM). A Horiba Jobin-YNON co-focal laser Raman system was used to obtain the Raman spectra, equipping a He–Ne laser with an excitation wavelength of 532 nm. Thermogravimetric analysis (TGA) was performed on a Mettler-Toledo TGA/SDTA851e Thermo Analyzer from room temperature to 800 °C at a rate of 5 °C min^−1^.

### Electrochemical measurements

Cyclic voltammetry (CV) and other tests were operated on a CHI 660E electrochemical station (Chenhua instrument Ltd., Shanghai) with a three-electrode cell system to measure the electrocatalytic activities of nanocomposites samples for methanol electro-oxidation reaction (MOR). Nanocomposites ribbons were used as the working electrodes directly. For powder samples, the catalyst suspensions were made by sonicating a mixture of 2.0 mg catalyst powders, 3.0 mg XC-72 carbon powders, 1.5 mL isopropanol and 0.5 mL Nafion solution (0.5 wt%) for 30 min. Then a proper amount of the catalyst suspension was dripped on pre-polished 4 mm diameter glassy carbon (GC) electrodes for electrochemical measurements. A bright Pt plate was used as counter electrode. And a Hg/HgO (1.0 M KOH) electrode (MMO) or a Hg/Hg_2_SO_4_ electrode (MSE) were used as the reference electrode, respectively. Electrolytes that we selected were 0.5 M KOH + 0.5 M methanol and 0.5 M H_2_SO_4_ + 0.5 M methanol, respectively. The scanning rates *v* of CVs were changed from 2 to 500 mV s^−1^. And the quasi steady-state anodic Tafel polarization analysis with the *v* of 1 mV s^−1^ was also measured in order to further study the sample catalytic activity for MOR. To evaluate the durability of the nanocomposite powders for MOR, the chronoamperograms (CAs) were recorded for a period of 3600 s at a fixed potential of 300 mV. All current densities were uniformed with the real surface of the samples.

## 3. Results and discussion

### Part 1: as-synthesized nanocomposite ribbons

#### A. Crystal structure and constituent of the ribbon samples

**Fig. 2:**
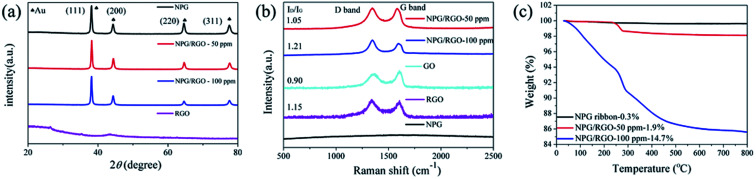
(a) XRD patterns, (b) Raman spectra and (c) TGA curves of nanocomposite ribbons (S0–S2) with varying *C*_GO_ (0–100 ppm), respectively.

As shown in Fig. 2a, the XRD patterns of NPG ribbon S0 and NPG/RGO composites S1 and S2 have four f.c.c. Au peaks (PDF no. 04-0784) with no obvious RGO peak, which is possibly due to the low content of the adsorbed GO. The lattice constant *a*_0_ and the preferred orientation factors *F* of (111) face *F*_(111)_ of samples are listed in Table 1. The *a*_0_ of ribbons increase with increasing the GO concentration (*C*_GO_) in dealloying process, and are larger than the *a*_0_ of standard Au (0.40786 nm). After dealloying, NPG has two stress regions:^57^ in the effect of tension regions, the bonds between Au atoms are easy to stretch, leading to a larger *a*_0_ (>0.40786 nm), and oppositely the compression regions result in a smaller *a*_0_ (<0.40786 nm). Thus, the tensile stress exists in NPG/RGO ribbons (Table 1). In addition, *F*_(111)_ values of ribbons are in the order: S1 > S2 > S0, which indicates a non-monotonic effect of adding GO on *F*_(111)_.

**Table 1:** The lattice constants *a*_0_ and the preferred orientation factors *F* of (111) face *F*_(111)_ of nanocomposite ribbons

Samples	S0	S1	S2
*a*_0_ (nm)	0.40799	0.40803	0.40804
*F*_(111)_	0.0838	0.247	0.174

Recently, NPG has been widely applied as the substrate of surface-enhanced Raman scattering (SERS) spectroscopy due to its large surface area and 3D bicontinuous porous structure.^50^ As a good substrate material, the Raman spectrum of S0 exhibits a smooth curve with no obvious peak (Fig. 2b). After adding RGO, the peaks at 1320 and 1620 cm^−1^ in the curves of S1 and S2 are corresponding to the breathing mode of aromatic rings of graphene (D band) and stretching mode of sp^2^ carbon (G band), respectively. The intensity ratio (*I*_D_/*I*_G_) of D-band and G-band usually stands for defect degree and a higher ratio exhibits more defects formed in the synthesizing process. As shown in Fig. 2b, the *I*_D_/*I*_G_ ratios of S1 and S2 samples are larger than 1.0, demonstrating that the RGO contains a large number of disordered carbon atoms, *i.e.* defects.^58^ In addition, the present Raman spectra indicate that RGO can be effectively combined with NPG.

To further explore the thermal stability of ribbons, the weight ratio of RGO in the NPG/RGO ribbons was determined by TGA under an atmosphere of compressed air. As shown in Fig. 2c, S0 presents an almost smooth line due to its slight weight loss with the removal of trace absorbed water at 245 °C, while the TGA curves of the S1 and S2 have a large loss and follow a similar trend in heating process. Except for the removal of absorbed water, the drastic weight loss starting at 245 °C of S1 and S2 is also related to the pyrolysis of some oxygen-containing functional groups. The mass loss at 500 °C can be attributed to burning of RGO.^53^ Here, in terms of the TGA results, the weight ratios of RGO in S1 and S2 are about 1.9, and 14.7%, respectively, confirming that the amount of RGO in NPG ribbons increases with increasing *C*_GO_ in dealloying process.

#### B. Microstructure of the as-synthesized nanocomposite

**Fig. 3:**
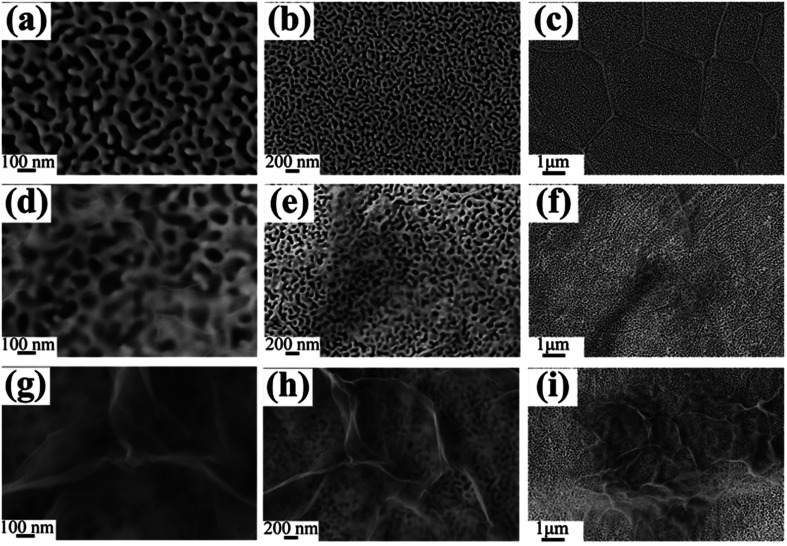
Plain-view SEM micrographs of S0 (a–c), S1 (d–f), and S2 (g–i), respectively.

SEM was operated to demonstrate whether the dealloying strategy to prepare NPG/RGO nanocomposite ribbons is effective or not. S0 has a uniform 3D bicontinuous interconnecting structure, with the pore size of 50–100 nm (Fig. 3a–c). The grain boundaries can be seen clearly in the low magnification image, with the grain size distributed in 5 μm (Fig. 3c). After adding GO solution in dealloying process, S1 has several transparent RGO layers in cicadas wing like shape anchored on the ligament/channel of NPG (Fig. 3d–f). Here, RGO sheets are not totally covering on the NPG structure. When *C*_GO_ increases to 100 ppm, the RGO layers of S2 are much thicker and darker than that of S1 (Fig. 3g–i). Three samples all exhibit the maze-like structure with the uniform pore size.

**Fig. 4:**
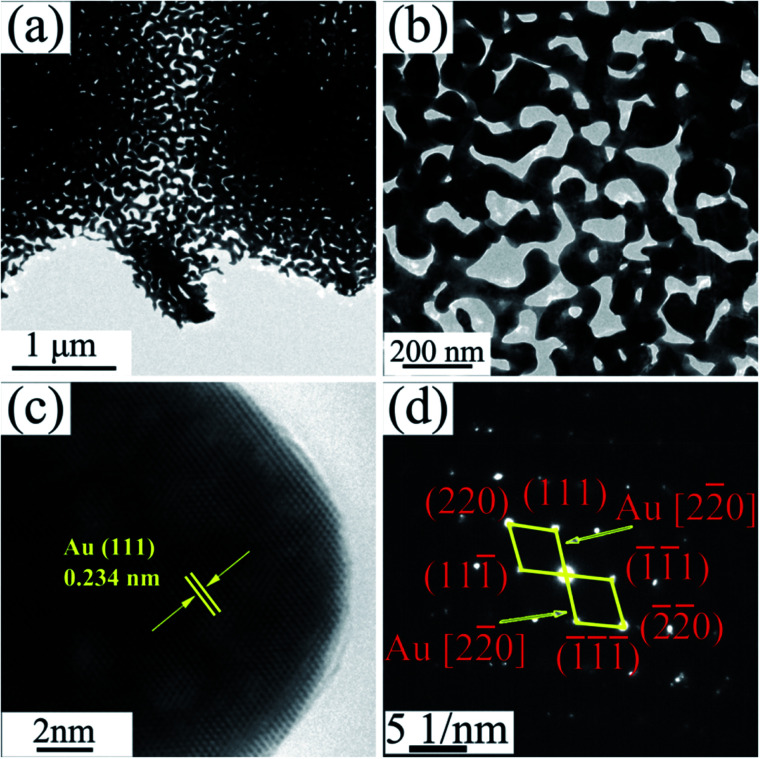
(a) TEM image of S0, (b) the corresponding enlarged image, (c) HRTEM image and (d) the corresponding SAED pattern.

The TEM images of NPG in Fig. 4a and b show that S0 has a ligament (dark-skeleton)-channel (bright region) structure with the pore size of 50–100 nm, being consistent with the SEM images in Fig. 3a–c. As shown in the HRTEM image (Fig. 4c), S0 has regular arranged lattice fringes with the same orientation, showing a single crystalline nature. And the lattice spacing (0.234 nm) of the single nanocrystal can be indexed as f.c.c. Au (111) reflection. In addition, the diffraction spots can be identified as Au (Fig. 4d), further verifying the single crystalline nature of S0.

**Fig. 5:**
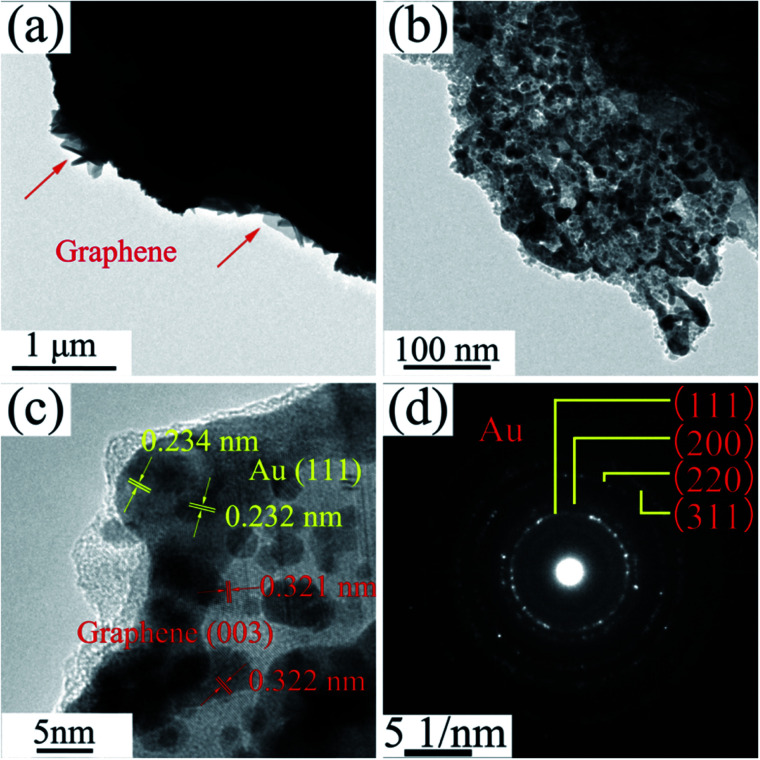
(a) TEM image of S1, (b) the corresponding enlarged image, (c) HRTEM image and (d) the corresponding SAED pattern.

The low magnification TEM image of S1 (Fig. 5a) shows that the RGO layers (as indicated in red arrow) can be observed in the edge of the NPG. S1 has a ligament/channel structure and a number of RGO layers covered on the NPG (top right corner of Fig. 5b). The regular arranged lattice fringes with two different lattice spacings in HRTEM image (Fig. 5c) are corresponding to Au and graphene phases, respectively. In addition, the polycrystalline diffraction rings in the corresponding SAED pattern (Fig. 5d) can be indexed as Au (111), (200), (220), and (311) peaks, which verifies that the nanocomposite S1 has a tremendous amount of Au nanocrystals.

#### C. Electro-oxidation of methanol catalyzed by nanocomposite ribbons

**Fig. 6:**
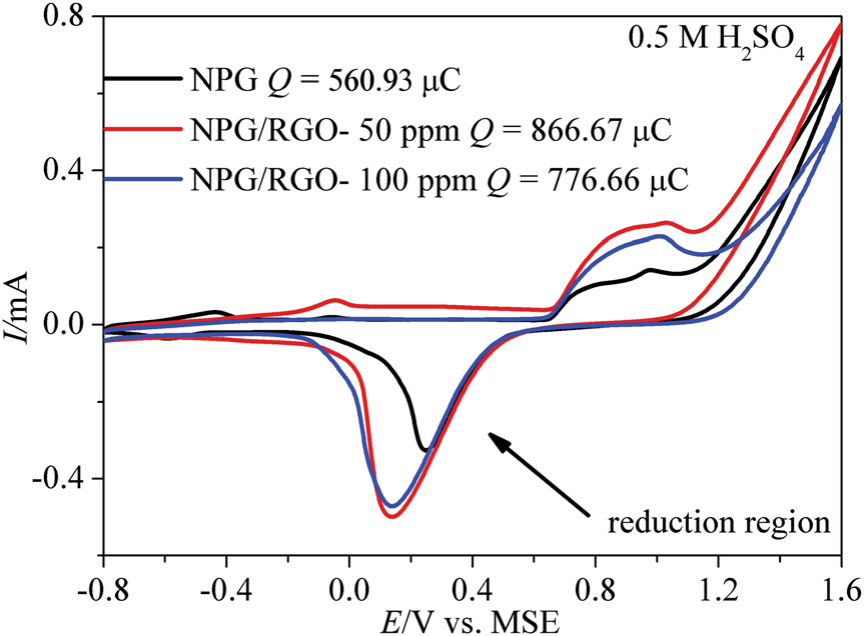
Cyclic voltammograms (CVs) of S0–S2 electrodes measured in 0.5 M H_2_SO_4_ solution. Scan rate *v*: 50 mV s^−1^.

**Fig. 7:**
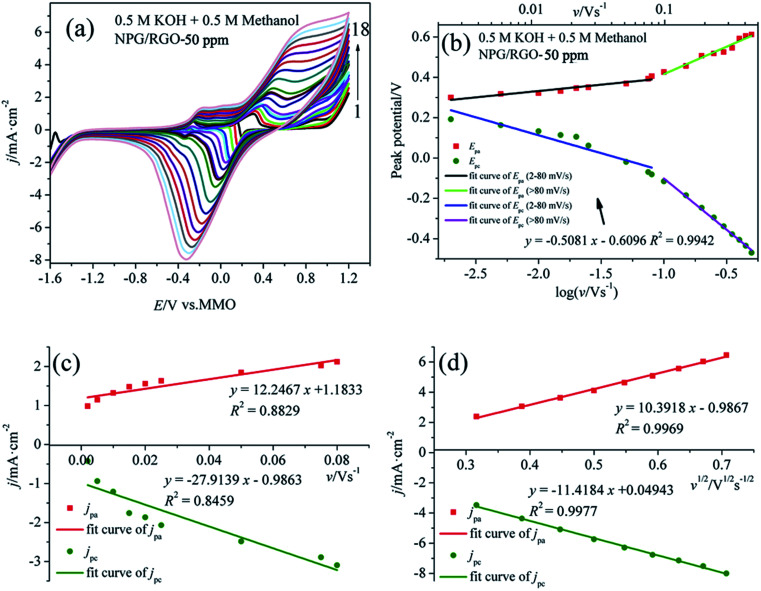
(a) CVs of S1 electrode in 0.5 M KOH + 0.5 M methanol solution at various scan rates *v*: (1) 0.002, (2) 0.005, (3) 0.01, (4) 0.015, (5) 0.02, (6) 0.025, (7) 0.05, (8) 0.075, (9) 0.08, (10) 0.1, (11) 0.15, (12) 0.2, (13) 0.25, (14) 0.3, (15) 0.35, (16) 0.4, (17) 0.45 and (18) 0.5 V s^−1^, (b) plot of *E*_p_*vs.* log *v* for CVs for anodic and cathodic peaks, (c) dependency of *j*_pa_ and *j*_pc_ on lower values of *v* (0.002–0.08 V s^−1^), and (d) on *v*^1/2^ at higher values of *v* (*v* > 0.08 V s^−1^).

**Fig. 8:**
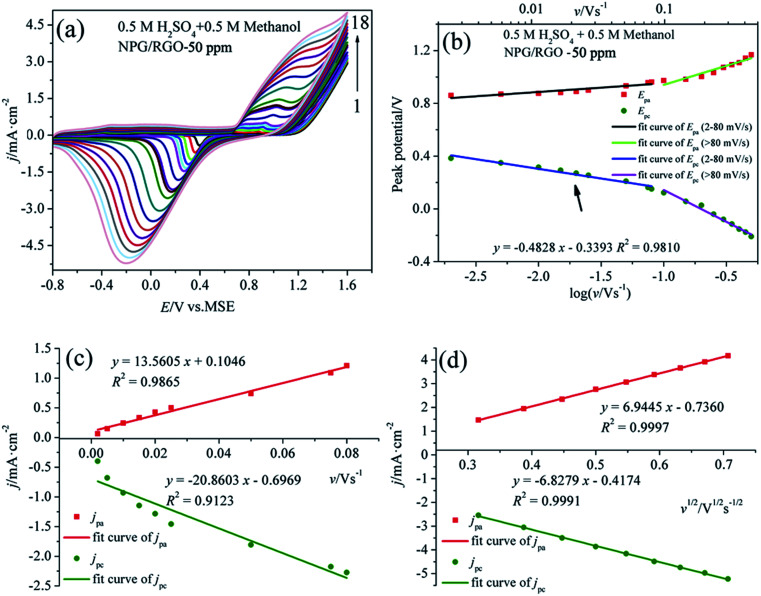
(a) CVs of S1 electrode in 0.5 M H_2_SO_4_ + 0.5 M methanol solution at various scan rates *v*, (b) plot of *E*_p_*vs.* log *v* for CVs for anodic and cathodic peaks, (c) dependency of *j*_pa_ and *j*_pc_ on lower values of *v* and (d) on *v*^1/2^ at higher values of *v*.

Fig. 6 exhibits the CVs of S0, S1, and S2 measured in 0.5 M H_2_SO_4_ solution. According to the oxygen adsorption measurement method, the real surface areas (*A*_real_) of S0, S1 and S2 electrodes are 1.45, 2.25, and 2.01 cm^2^, respectively. And the surface roughness factors *r*_f_ (denoted as the ratio between *A*_real_ and geometry area *A*_geom_, 0.2 cm^2^) of S0, S1 and S2 are 7.25, 11.25, and 10.05, respectively. Here, S1 has a maximal *A*_real_. The electro-catalytic activities like CVs of ribbon electrodes for MOR were shown in Fig. 7, 8 and S1–S4† at *v* = 2–500 mV s^−1^ in 0.5 M KOH + 0.5 M and 0.5 M H_2_SO_4_ + 0.5 M methanol solutions. Apparently, increasing the *v* could increase the current density of CVs of samples.

Generally, the CVs test is a major method to analyze the electrochemical behavior of an electrode. More analysis details can be obtained from our former reports.^32^ In order to better understand the mechanism of MOR more clearly, independent CV curve of S1 electrode tested in alkaline solution is shown in Fig. S5.† Briefly, MOR on Au electrode occurs in two regions with different mechanisms.^12,59,60^ As shown in Fig. S5,† in the lower potential region from −0.6 to 0.4 V *vs.* MMO, MOR commences in the surface of oxide-free electrode. The subpeak at ∼−0.3 V *vs.* MMO (peak 1) is attributed to the emergence of the “pre-oxidation species” (eqn (1)), and *λ* (0–1) represents the charge-transfer coefficient.^61^

1 Au + OH^−^ − *λ*e^−^ → Au–OH_ads_^(1−*λ*)−^

With increasing potential, Au–OH_ads_^(1−*λ*)−^ is oxidized to AuO, and formate is the mainly product of MOR through a four-electron-transfer reaction as following:^12,60^ We can observe the appearance of peak 2 in Fig. S5.†

2 Au–OH_ads_^(1−*λ*)−^ + OH^−^ − (2 − *λ*)e^−^ → AuO + H_2_O

3 CH_3_OH + 5OH^−^ = HCOO^−^ + 4H_2_O + 4e^−^

As potential surpasses 0.45 V *vs.* MMO, the oxidation of methanol could be weakened by the transformation of Au–OH_ads_^(1−*λ*)−^ to AuO, corresponding to the current density decreases. Benefiting from the re-exposed of the fresh Au surface oxide, the oxidation of methanol to carbonate emerged on the gold oxides *via* a six-electron-transfer reaction (eqn (4)), leading to an obvious increment of anodic current densities for MOR (peak 3).^12,62^

4 CH_3_OH + 8OH^−^ = CO_3_^2−^ + 6H_2_O + 6e^−^

The step by step reaction is helpful for the oxidation of intermediates at high potential, which can eliminate the possible catalyst poisoning.^17^ In the following negatively sweeping, MOR re-starts according to eqn (3) and is accompanying with the removal of dense AuO; hence, it increases the absolute values of the corresponding oxidation current densities (peak 4).^12,62^ The small peak at around −0.30 V *vs.* MMO (peak 5) is related with the oxygen reduction reaction in the solution. Fig. 7b shows the *E*_pa_*vs.* log *v* and *E*_pc_*vs.* log *v* curves deduced from the measured CVs. Apparently, the potential difference Δ*E*_p_ (*E*_pa_ − *E*_pc_) increases with increasing *v*, suggesting the charge transfer kinetic limitation in the reaction.^63^

It is well known that the electron-transfer coefficient (*α*), and apparent charge-transfer rate constant *k*_s_ (s^−1^) can be obtained from the Laviron theory (eqn (S3)–(S5)†) if Δ*E*_p_ > 0.2/*n* V.^64^ We selected the curve of *E*_pc_*vs.* log *v* and obtained *α* and *k*_s_ (0.058 and 0.053 s^−1^), which indicates that the electrochemical process is the major rate limiting step according to the former reports.^63,65^ Usually, a larger *k*_s_ could represent a faster oxidation process. In addition, the linear relationship between *v* and peak current densities in 2–80 mV s^−1^ can be observed in Fig. 7c, which is ascribed to the activity of the surface redox couple.^66^ Here, the surface coverage Γ* (mol cm^−2^) of the redox species, *i.e.*, the immobilized active species, is deduced according to eqn (S6).†^67,68^ The calculated Γ* of S1 electrode is 5.10 × 10^−6^ mol cm^−2^ after averaging the cathodic and anodic current densities. On the other hand, when *v* > 80 mV s^−1^, there is a good linear relation between the current densities of anodic/cathodic peaks and *v*^1/2^ (Fig. 7d), leading to a diffusion control step.^66^

Fig. 8a shows the CVs of S1 electrode in 0.5 M H_2_SO_4_ + 0.5 M methanol solution at different *v*, where the “pre-oxidation species” is absent due to the low OH^−^ concentration in the acid electrolyte. Apparently, a linear relationship between *E*_p_ and log *v* can be observed in Fig. 8b. Using the above mentioned method, the deduced Γ*, *α*, and *k*_s_ of samples in two kinds of electrolytes are summarized in Table 2. In the presence of OH^−^, the Γ* and *k*_s_ of electrodes are higher than those in the absence of OH^−^, suggesting that OH^−^ plays an important role in MOR.^17^ Moreover, the Γ* and *k*_s_ of S1 are higher than those of S2 and S0. Meanwhile, the current densities of oxidation and reduction peaks of S1 are higher than those of the other samples (Figs. 7, 8, S1–S4), suggesting that the activity for MOR of S1 is the highest. For comparison, the CVs of electrodes in acid and alkaline solutions without methanol are exhibited in Fig. S6.† The *E*_p_*vs.* log *v* of ribbon electrodes is given in Fig. S7.† Fig. S8† shows the CVs of S1 electrode with 50 cycles in alkaline and acid solutions with the scan rate *v* of 50 mV s^−1^. It can be found that with increasing the cycle, the current densities of oxidation peaks increase, indicating that electrode exhibits a good durability.^69^

**Table 2:** The surface coverage of the redox species Γ*, electron-transfer coefficient *α* and apparent charge-transfer rate constant between the electrode and the surface-deposited layer *k*_s_ obtained from CVs of ribbon electrodes with *v* = 2–80 mV s^−1^ in alkaline and acid solutions, respectively

Solutions	Samples	Γ* (10^−6^ mol cm^−2^)	*α*	*k*_s_ (s^−1^)
0.5 M KOH + 0.5 M methanol	S0	1.67	0.089	0.017
	S1	5.10	0.058	0.053
	S2	4.75	0.071	0.044
0.5 M H_2_SO_4_ + 0.5 M methanol	S0	1.60	0.083	0.0064
	S1	4.07	0.061	0.014
	S2	3.44	0.073	0.0098

**Fig. 9:**
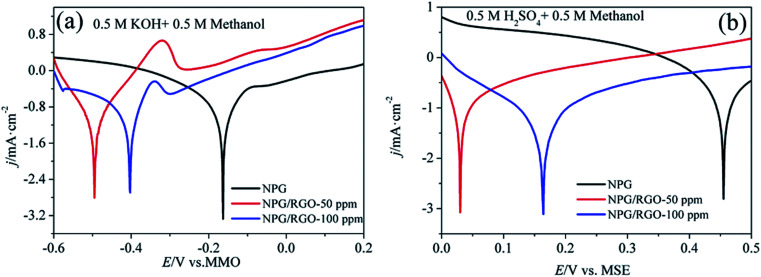
Tafel curves of S0–S2 electrodes in (a) 0.5 M KOH + 0.5 M methanol and (b) 0.5 M H_2_SO_4_ + 0.5 M methanol solutions, respectively. *v*: 1 mV s^−1^.

Tafel polarization curves of ribbon electrodes in two kinds of electrolytes are shown in Fig. 9. The measured voltage range in alkaline acid solutions were −0.6–0.2 V *vs.* MMO, and 0–0.5 V *vs.* MSE, respectively. Several Tafel parameters such as the Tafel slopes *b*, exchange current densities *j*_0_, and corrosion potential *E*_corr_ can be acquired according to eqn (S7)–(S9),†^70,71^ which are listed in Table 3. Moreover, there are current plateaus in the anodic parts in two solutions (Fig. 9b), demonstrating that in this region the charge transfer control is the leading step.^72,73^ The deduced *b*, *j*_0_ and *E*_corr_ confirm the highest activity of S1 electrode for MOR.

**Table 3:** The corrosion potential *E*_corr_ (V), anodic slope *β*_a_ (mV dec^−1^), and exchange current density *j*_0_ (mA cm^−2^) obtained from Tafel curves of ribbon electrodes in alkaline and acid solutions, respectively

Solutions	Samples	*E*_corr_ (V)	*β*_a_ (mV dec^−1^)	*j*_0_ (mA cm^−2^)
0.5 M KOH + 0.5 M methanol	S0	−0.162	160.2	3.25
	S1	−0.495	38.8	8.01
	S2	−0.403	87.2	6.74
0.5 M H_2_SO_4_ + 0.5 M methanol	S0	0.455	186.0	2.67
	S1	0.030	77.5	7.25
	S2	0.164	89.3	5.37

### Part 2: as-synthesized nanocomposite powders

Besides the nanocomposite ribbon results in Part 1, the effect of GO on the dealloying process of Al_2_Au powders was further investigated.

#### A. Crystal structure and constituent of powders samples

**Fig. 10:**
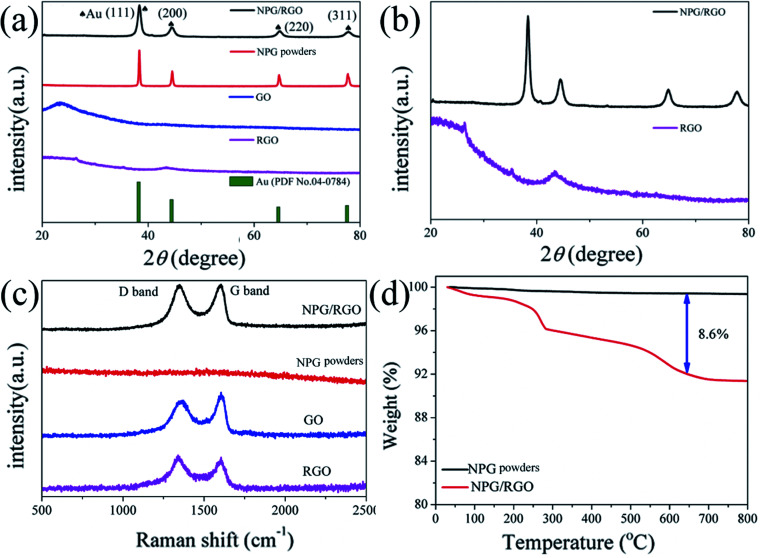
(a) XRD patterns of nanocomposite powders P0, P1, GO, and RGO, (b) enlarged XRD patterns of P1 and RGO, (c) Raman spectra of P0, P1, GO, and RGO and (d) TGA curves of P0 and P1, respectively.

The structure details such as XRD patterns and TEM images of powder precursor crushed from ingot are displayed in Fig. S9 and S10.† The XRD patterns and Raman spectra of P0, P1, GO, and RGO are shown in Fig. 10 together with the TGA curves of P0 and P1. The Au peaks of P0 are sharper than P1 (Fig. 10a and b). The *a*_0_ of P0 and P1 powders (0.40769 and 0.40667 nm) are smaller than that of the standard Au (0.40786 nm). Hence, the compressive stress is dominant in powder sample.^32,57^ In addition, the *F*_(111)_ values of P0 and P1 powders are 0.006 and 0.0187, respectively. Accompanying with the reduction of GO, there is no obvious GO peaks in XRD patterns of P1. The enlarged XRD patterns show that the broaden diffractions of P1 and RGO diffractions are overlapped (Fig. 10b). The broadened peaks of P1 are probably attributed to the RGO.

The intensity of D band of P1 is slightly higher than that of G band in Raman spectrum (Fig. 10c), indicating that the RGO in P1 contains a large number of disordered carbon atoms and defects. RGO can be effectively combined with the nanostructure of NPG according to the Raman spectra results. The mass loss at around 500 °C is probably attributed to burning of RGO (Fig. 10d)^53^ and the weight percent of RGO in P1 samples are about 8.6%.

#### B. Microstructure of the as-synthesized nanocomposite powders

**Fig. 11:**
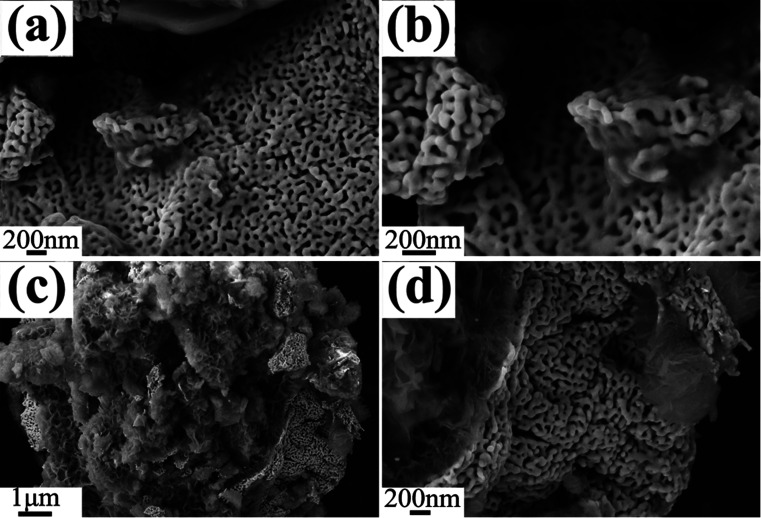
Plain-view SEM micrographs (a and c) of P1, (b) and (d) are the corresponding magnification images of (a) and (c).

As shown in Fig. S11,† SEM images of P0 is similar with S0 except for the certain ligament coarsening. SEM images of P1 show a uniform maze-like structure with the attachment of several transparent RGO layers, accompanying with broadening of ligament (Fig. 11a and b). As shown in Fig. 11c and d, the sphere-structure NPG is surrounded by the RGO clusters. During milling the precursor ingot, RGO sheets intersperse randomly on the spherical NPG powders to reduce their surface energy and form an armour structure. We suppose that the unique structure plays a facilitating role in the electro-catalytic behavior of NPG.

**Fig. 12:**
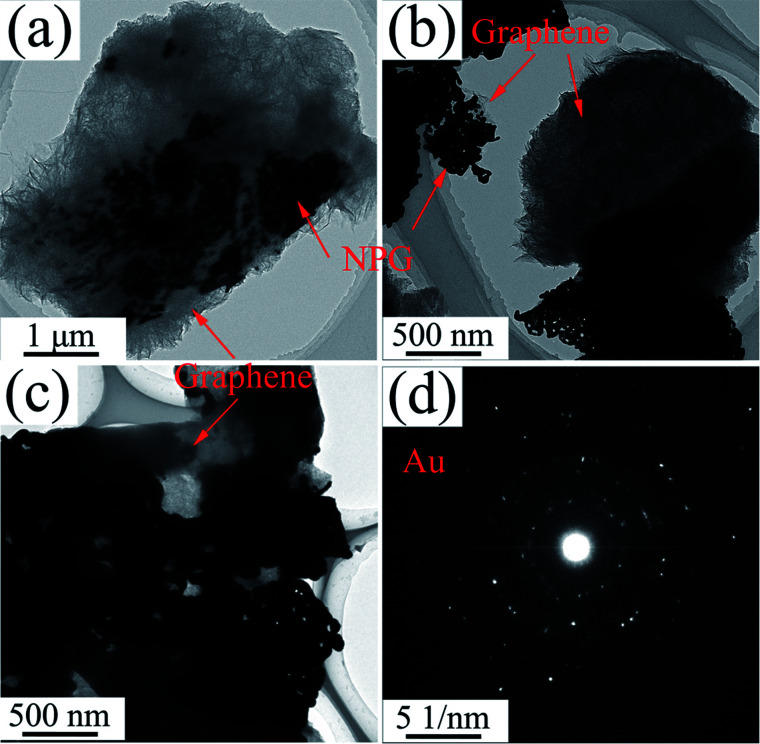
(a–c) TEM images and (d) SAED image of P1.

The typical TEM images of P0 are analogous to S0 and not shown here. In TEM images of P1 (Fig. 12a–c), gray RGO layers are combined with the dark NPG in a ligament/channel structure. The corresponding SAED pattern (Fig. 12d) consists of polycrystalline diffraction rings and is consistent with those in Fig. 5d, which verifies that the nanocomposite powder sample is also composed of a tremendous amount of Au nanocrystals.

#### C. Electro-oxidation towards methanol of as-synthesized nanocomposites samples

**Fig. 13:**
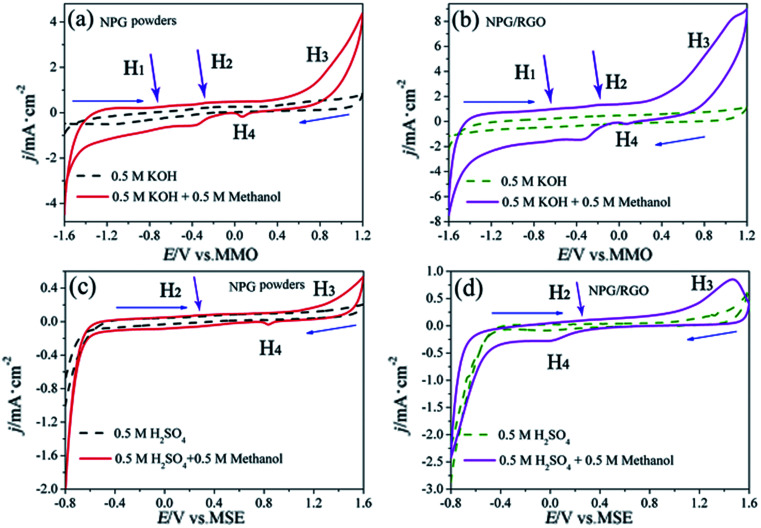
CVs of P0 and P1 electrodes in 0.5 M KOH + 0.5 M methanol solution (solid lines in (a) and (b)), 0.5 M KOH solution (dash lines in (a) and (b)), 0.5 M H_2_SO_4_ + 0.5 M methanol solution (solid lines in (c) and (d)), and 0.5 M H_2_SO_4_ solution (dash lines in (c) and (d)), respectively, scan rates *v*: 50 mV s^−1^.

As shown in CVs of P0 and P1 in alkaline methanol solution (Fig. 13a and b), the oxidation peaks H_1_ and H_2_ in the low potential regions (−0.6–0.4 V *vs.* MMO) represent the formation of “pre-oxidation species” (eqn (1)) and the oxidation of methanol to formates (eqn (2) and (3)), respectively. When potential is higher than 0.45 V *vs.* MMO, the formation of peak H_3_ is associated to the methanol oxidation *via*eqn (4), leading to the obvious increase of the anodic current density. An obvious reduction peak H_4_ can be observed in the negatively-going scan, which is related to the removal of dense AuO layer, and further stimulate the eqn (1) reaction.

**Fig. 14:**
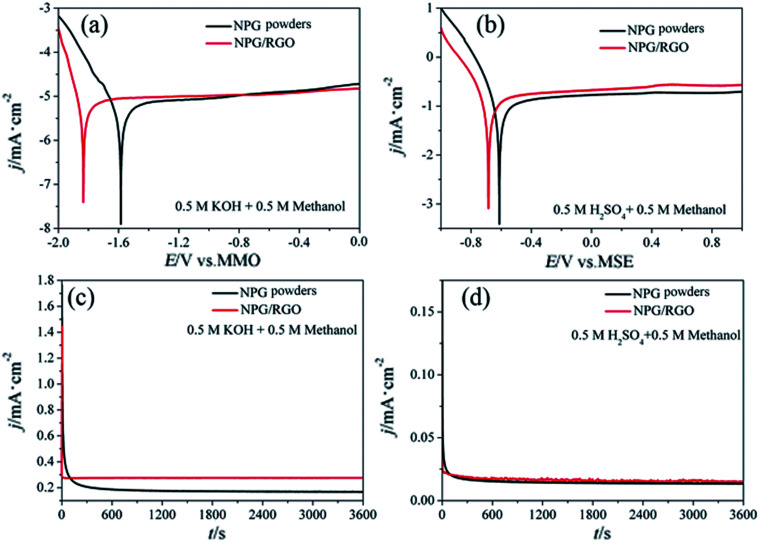
Tafel curves of P0 and P1 electrodes in (a) 0.5 M KOH + 0.5 M methanol and (b) 0.5 M H_2_SO_4_ + 0.5 M methanol solutions, respectively. *v*: 1 mV s^−1^. Chronoamperograms (CAs) were recorded for a period of 1000 s at 300 mV in (c) 0.5 M KOH + 0.5 M methanol and (d) 0.5 M H_2_SO_4_ + 0.5 M methanol solutions, respectively.

The MOR behavior of P0 and P1 electrodes in acid electrolyte is similar to alkaline solution except for H_1_ peaks (Fig. 13c and d). In both cases, the current densities of P1 electrode are higher than that of P0 electrode. Tafel curves of P0 and P1 electrodes are shown in Fig. 14. According to eqn (S7)–(S9),† the parameters obtained from Tafel curves of electrodes are listed in Table 4. These parameters further verify that P1 has a higher catalytic activity for MOR than P0 electrode, consisting with the CVs results. The stability of a catalyst is an important requirement for its practical applications. In order to evaluate the stability of P0 and P1 for MOR, chronoamperograms (CAs) are recorded for a period of 3600 s at a fixed potential of 300 mV in alkaline (Fig. 14c) and acid solutions (Fig. 14d), respectively. For P0 and P1 electrodes, a rapid current decay appears within the limited seconds, accompanying by a slow drop until it reaches a smooth state. According to the literature,^74^ the formation of double layer capacitance leads to the fast drop at first, and the following decay of the CAs is related to the accumulation of intermediate species during the process of methanol decomposition. The current density gradually reaches a quasi-equilibrium steady state with the further polarization. Moreover, the current density for P1 in both solutions is higher than that for P0 electrode in the whole process, indicating that P1 exhibits a relatively higher activity for MOR. Fig. S12† also presents SEM images of P1 electrode after electrochemical tests, implying the marvelous structural stability of P1 electrode.

**Table 4:** The corrosion potential *E*_corr_ (V), anodic slope *β*_a_ (mV dec^−1^), and exchange current density *j*_0_ (mA cm^−2^) obtained from Tafel curves of P0 and P1 electrodes in alkaline and acid solutions, respectively

Solutions	Samples	*E*_corr_ (V)	*β*_a_ (mV dec^−1^)	*j*_0_ (mA cm^−2^)
0.5 M KOH + 0.5 M methanol	P0	−1.585	124.9	4.64
	P1	−1.834	78.2	5.53
0.5 M H_2_SO_4_ + 0.5 M methanol	P0	−0.612	166.8	4.01
	P1	−0.684	90.4	5.11

### Part 3: catalytic mechanism of NPG/RGO nanocomposites

By adding moderate GO in dealloying process, we have obtained the NPG/RGO nanocomposite ribbons and powders with high activity for MOR. The high activity for MOR of NPG/RGO nanocomposites can be explained as follows.

Firstly, the activity for MOR of samples is in the following order: S1 > S2 > S0 and P1 > P0. The *F*_(111)_ of ribbon samples ranks as S1 > S2 > S0 (Table 1) and the *F*_(111)_ of powder samples is P1 > P0. According to the report,^75^ the anodic current density of Au (111) is the highest compared to those of Au (110) and (100), demonstrating that under the same conditions, Au (111) is more active than the other faces. This result can also be proved in our former works.^32,76^ We speculated that the formation of the low-index crystal plane (111) can be effected by the reduction of GO. The specific formation mechanism can be further investigated.

Secondly, raising *r*_f_ of a catalyst can improve its catalytic activity due to two facts:^18^ (1) the *A*_real_ of nanocomposites can be extended by the attachment of RGO sheets and supply more active sites for MOR; (2) the interconnected nanoporous channels of NPG can provide a faster pathway for transfering charge/ions with a larger pore size.

Finally, S0 has a single crystal structure with a regular crystal face arrangement (Fig. 4), but S1 and P1 bear a polycrystal structure with a tremendous amount of Au nanocrystals as well as a few RGO (Fig. 5 and 12). More grain boundaries and defects exist in the nanocrystal structure of S1 and P1, compared to the single crystal. Also, according to XRD results (Fig. 2a and 10a), tensile stresses and compressive stresses exist in ribbon and powder samples, respectively. Material microstress can facilitate the catalytic activity of nanocomposite samples in the catalysis process.^57,77^ Raman results show that RGO contains a large number of disordered carbon atoms, *i.e.* a large amount of defects.^58^ The defects of RGO can help ions/electrons to transfer in electrochemical reaction process. In addition, according to the report of Bäumer *et al.*,^30^ the traced residual Al impurities undetected by XRD are crucial for the excellent oxidative activity of NPG and could also contribute to the activity of Au nanostructures. Grain boundary, stress, RGO defects and residual Al atoms are four kinds of defects in NPG/RGO ribbon and powder nanocomposites, which can benefit the catalytic activity of NPG.^78^

In short, in the cooperation of the participation of RGO sheets and unique ligament/channel nanostructure of NPG, NPG/RGO ribbon and powder nanocomposites have a better catalytic activity for MOR than NPG. Adding GO in dealloying process could be one new method to obtain the NPG materials with the excellent catalytic activity.

## 4. Conclusions

(1) The nanoporous gold and reduced oxidized graphene (NPG/RGO) nanocomposites in ribbon shape were successfully prepared by dealloying Al_2_Au_1_ ribbons in 10 wt% HCl solution with various GO concentration (*C*_GO_ = 0, 50, and 100 ppm), which were labeled as S0, S1, and S2, respectively. The RGO nanosheets of S1 and S2 are anchored on the surface of NPG in a cicadas wing like shape.

(2) With increasing the *C*_GO_, the lattice constant *a*_0_, and nanograin boundary density of 3D bicontinuous NPG and the thickness of RGO tend to increase. The catalytic activity of S1 for methanol electro-oxidation reaction (MOR) is the highest due to the optimized NPG structure and the role of conductive agent and buffer layer played by RGO.

(3) The NPG/RGO nanocomposites in powder shape were also successfully prepared by dealloying the Al_2_Au powders in 10 wt% HCl solution with *C*_GO_ = 0 and 50 ppm (labeled as P0 and P1). The catalytic activity for MOR of P1 is higher than P0 and has a marvelous structural stability after electrochemical tests. These results supply a new method for us to obtain the nanoporous gold with excellent catalytic activity through adding GO in dealloying process.

## Conflicts of interest

There are no conflicts to declare.

## Supplementary Material

RA-010-C9RA09821F-s001
