# Supplementary material for: Dealloyed porous gold anchored by in situ generated graphene sheets as high activity catalyst for methanol electro-oxidation reaction
Source: RSC Adv. 2020 Jan 9;10(3):1666–78. doi: 10.1039/c9ra09821f (PMC9047550; doi:10.1039/c9ra09821f)
Supplement: RA-010-C9RA09821F-s001 [file RA-010-C9RA09821F-s001.pdf]

Electronic Supplementary Material (ESI) for RSC Advances.
This journal is © The Royal Society of Chemistry 2019

Supporting Information

Dealloying porous gold anchored by in situ generated graphene sheets as high activity catalyst for methanol electro-oxidation reaction

Hui Xu, *[a,b] Shuai Liu,[b] Xiaoliang Pu, [b] Kechang Shen,[c] Laichang Zhang,[d] Xiaoguang Wang,[e] Jingyu Qin,[b] and Weimin Wang*[b]

[a] State Key Laboratory for Modification of Chemical Fibers and Polymer Materials, International Joint Laboratory for Advanced Fiber and Low-dimension Materials, College of Materials Science and Engineering, Donghua University, Shanghai 201620, China

[b] Key Laboratory for Liquid-Solid Structural Evolution and Processing of Materials, Ministry of Education, Shandong University, Jinan 250061, China

[c] Ulsan Ship and Ocean College, Ludong University, Yantai 264025, China

[d] School of Engineering, Edith Cowan University, 270 Joondalup Drive, Joondalup, Perth, WA6027, Australia

[e] Laboratory of Adv. Mater. & Energy Electrochemistry, Taiyuan University of Technology, Taiyuan 030024, China

*Corresponding author.
E-mail address: xuhui199278@dhu.edu.cn; weiminw@sdu.edu.cn

1. The specific calculation methods of lattice constant a_0 and preferred orientation factors F.

The lattice constant a_0 of samples were calculated by the extended Bragg equation:

$$a_0 = \frac{\lambda}{2\sin\theta}\sqrt{h^2 + k^2 + l^2}, \qquad \text{(S1)}$$

where λ is the radiation wavelength (λ = 0.1542 nm); θ is the diffraction angle; and (h, k, l) are the crystal plane indices. The preferred orientation factors F of (111) face of samples were also investigated by the Lotgering method:

$$F = \frac{P - P_0}{1 - P_0}, P = \frac{\sum I_{(h00)}}{\sum I_{(hkl)}} \text{ and } P_0 = \frac{\sum I_{0(h00)}}{\sum I_{0(hkl)}}, \qquad \text{(S2)}$$

where $I_{(h00)}$ and $I_{(hkl)}$ are the integral intensities of diffraction peaks for composites samples and randomly oriented samples, respectively; and P and P_0 are the ratios of integral intensities of (h00) planes to those of all (hkl) planes for composites samples and randomly oriented samples, respectively.

2. The specific calculation methods of the electron-transfer coefficient α, charge-transfer rate constant k_s, and surface coverage Γ.*

In the case of $\Delta E_p > 0.2/n$ V, where n is the number of exchanged electrons, the electrochemical parameters such as electron-transfer coefficient α, and apparent charge-transfer rate constant between the electrode and the surface-deposited layer k_s (s^{-1}) can be calculated by the following equations according to the Laviron theory (equations (S3)~(S5)):

$$E_{pa} = E^0 + \frac{RT}{(1-\alpha)nF}\ln\left[\frac{RTk_s}{(1-\alpha)nF}\right] + \frac{RT}{(1-\alpha)nF}\ln v \qquad \text{(S3)}$$

$$E_{\text{pc}} = E^0 + \frac{RT}{\alpha nF}\ln\left[\frac{RTk_{\text{s}}}{\alpha nF}\right] - \frac{RT}{\alpha nF}\ln v \quad \text{(S4)}$$

$$\ln k_{\text{s}} = \alpha\ln(1-\alpha) + (1-\alpha)\ln\alpha - \ln\frac{RT}{nFv} - \frac{\alpha(1-\alpha)nF\Delta E_{\text{p}}}{RT} \quad \text{(S5)}$$

where E_{pa}, E_{pc} and E^0 are the anodic peaks, cathodic peaks, and standard potentials (V), respectively.

The surface coverage Γ^* (mol/cm^2) of the redox species can be calculated from the slopes of j_{p}-v line and equation (S6):

$$j_{\text{p}} = \frac{n^2F^2Av\Gamma^*}{4RT} \quad \text{(S6)}$$

3. The specific calculation methods of the Tafel slopes b, exchange current densities j_0, and corrosion potential E_{corr}.

Based on the Tafel theory (equation (S7)~(S9)), the relation between the overpotential η and the current density j is:

$$\eta = a + b\log j \quad \text{(S7)}$$

$$a = -\frac{2.303RT\log j_0}{\alpha mF} \quad \text{(S8)}$$

$$b = \frac{2.303RT}{\alpha mF} \quad \text{(S9)}$$

where j_0 is the exchange current density, α is the anodic transfer coefficient, m is the number of transferred electrons and b is the Tafel slope.

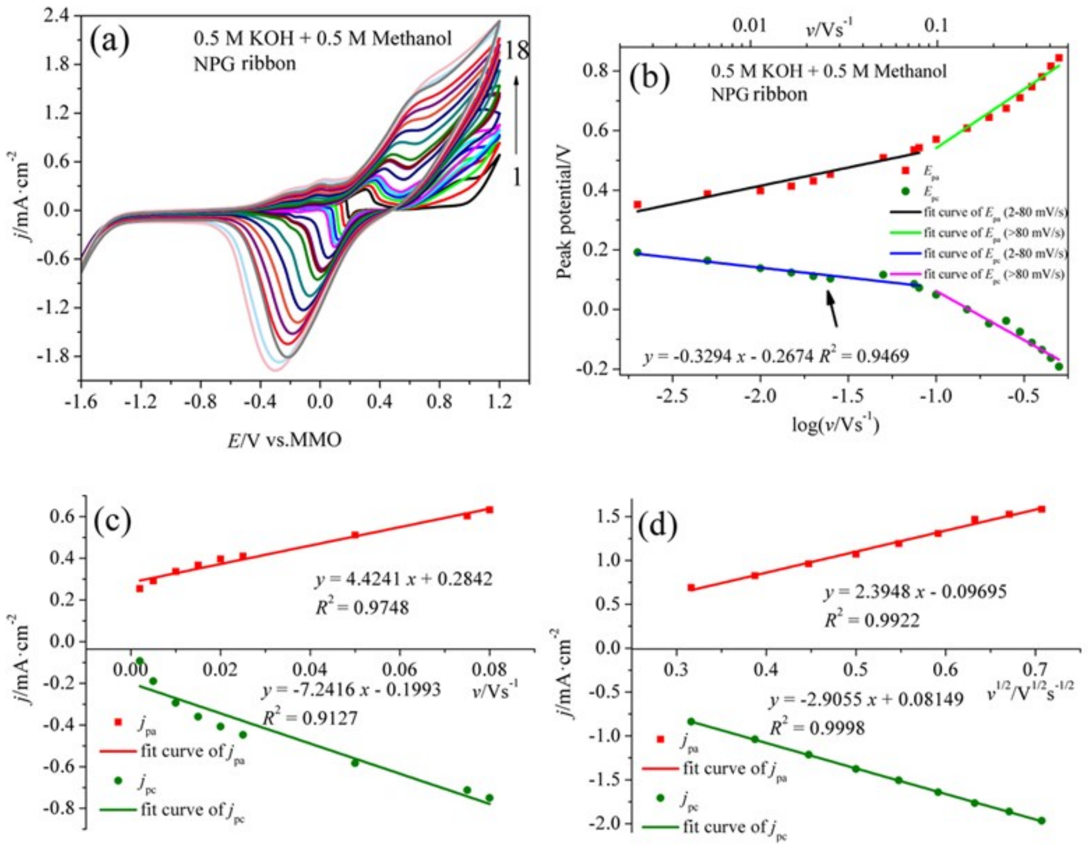

Fig. S1. (a) CVs of S0 electrode in 0.5 M KOH + 0.5 M methanol solution at various scan rates v: (1) 0.002, (2) 0.005, (3) 0.01, (4) 0.015, (5) 0.02, (6) 0.025, (7) 0.05, (8) 0.075, (9) 0.08, (10) 0.1, (11) 0.15, (12) 0.2, (13) 0.25, (14) 0.3, (15) 0.35, (16) 0.4, (17) 0.45 and (18) 0.5 V s^{-1}, (b) plot of E_p vs. log$v$ for CVs for anodic and cathodic peaks, (c) dependency of j_{pa} and j_{pc} on lower values of v (0.002-0.08 V s^{-1}) and (d) on $v^{1/2}$ at higher values of v (v > 0.08 V s^{-1}).

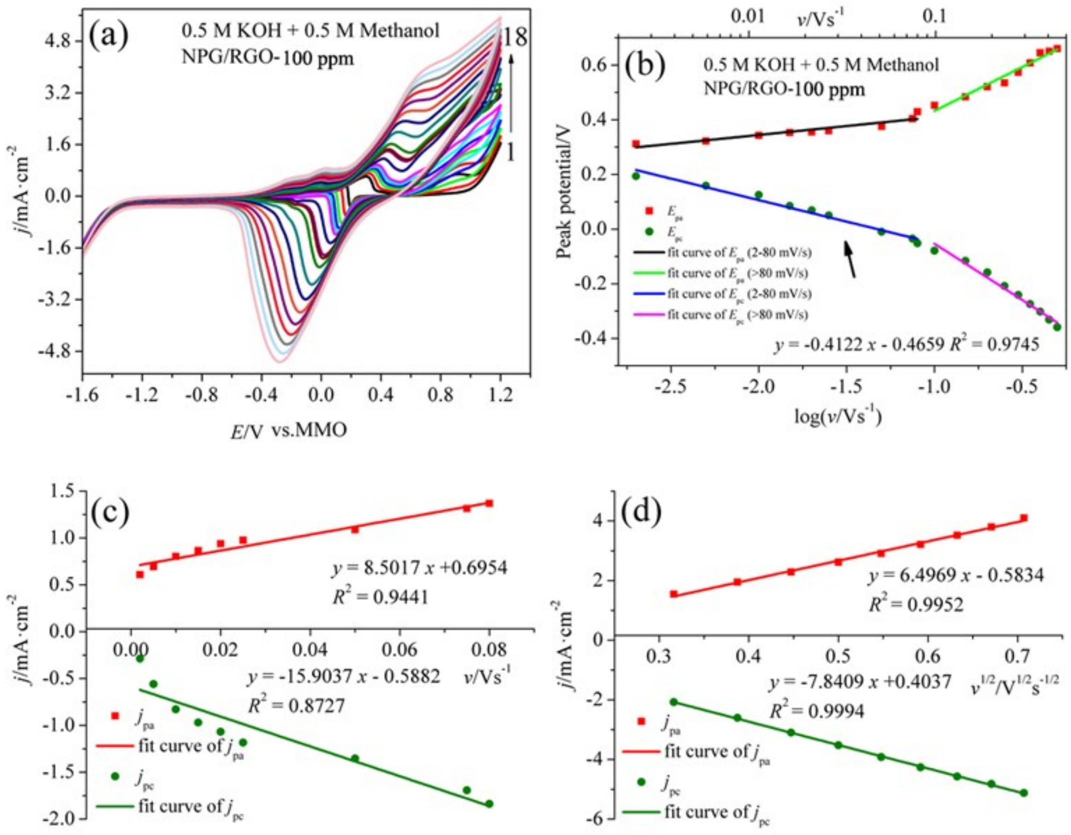

Fig. S2. (a) CVs of S2 electrode in 0.5 M KOH + 0.5 M methanol solution at various scan rates v, (b) plot of E_p vs. log$v$ for CVs for anodic and cathodic peaks, (c) dependency of j_{pa} and j_{pc} on lower values of v and (d) on $v^{1/2}$ at higher values of v.

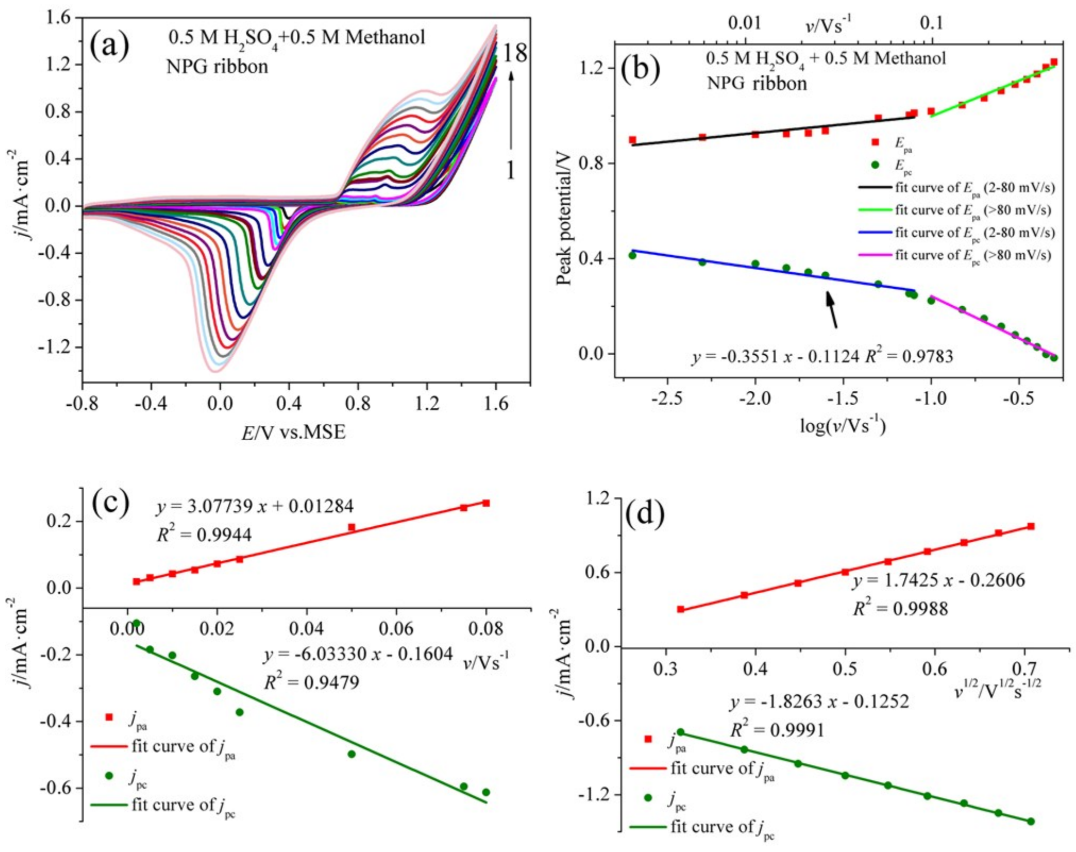

Fig. S3. (a) CVs of S0 electrode in 0.5 M H_2SO_4+ 0.5 M methanol solution at various scan rates v, (b) plot of E_p vs. log$v$ for CVs for anodic and cathodic peaks, (c) dependency of j_{pa} and j_{pc} on lower values of v and (d) on $v^{1/2}$ at higher values of v.

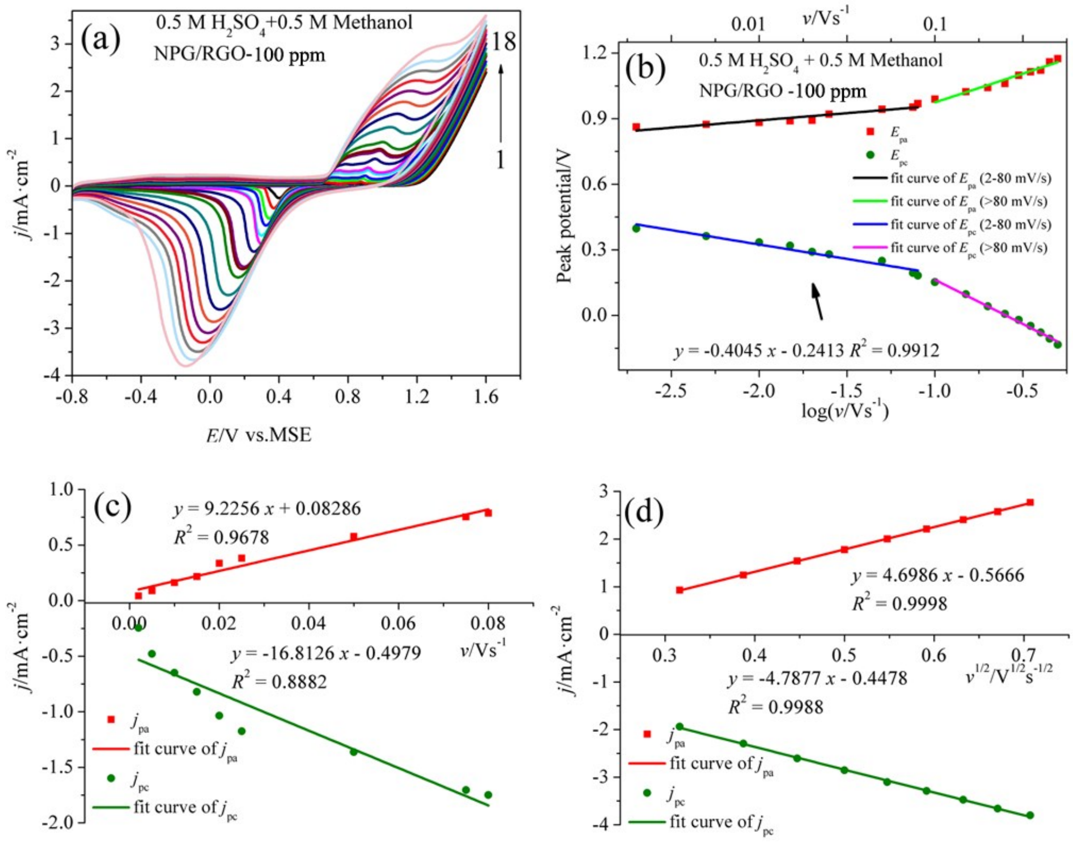

Fig. S4. (a) CVs of S2 electrode in 0.5 M H_2SO_4+ 0.5 M methanol solution at various scan rates *v*, (b) plot of E_p vs. log*v* for CVs for anodic and cathodic peaks, (c) dependency of j_{pa} and j_{pc} on lower values of *v* and (d) on $v^{1/2}$ at higher values of *v*.

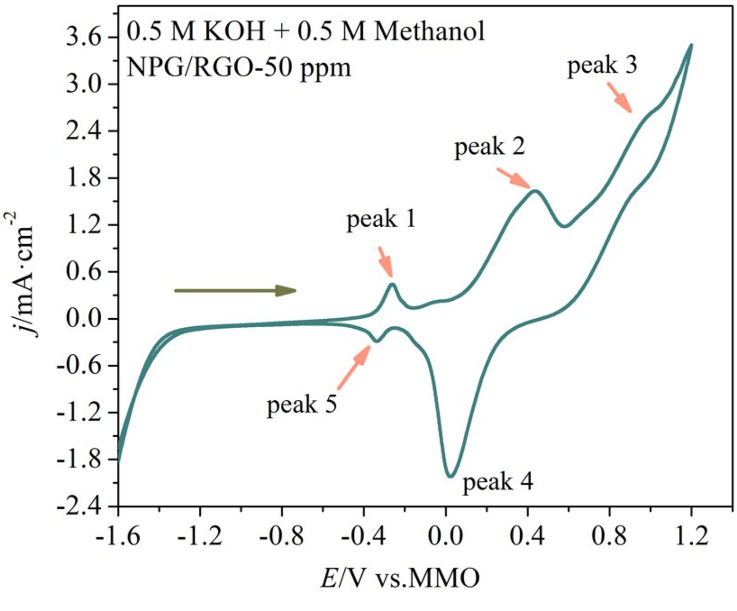

Fig. S5. Cyclic voltammograms (CVs) of S1 electrodemeasured in 0.5 M KOH + 0.5

M methanol solution. Scan rate v: 50 mV/s.

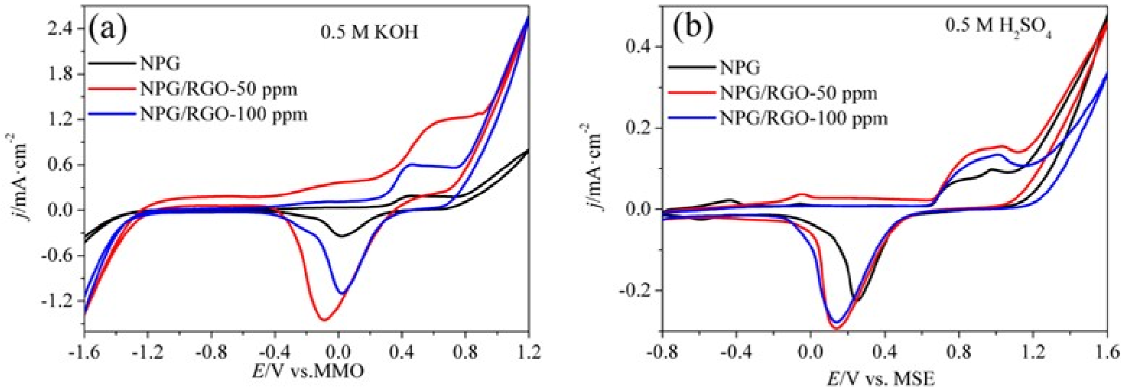

Fig. S6. CVs of S0~S2 electrodes in (a) 0.5 M KOH solution and (b) 0.5 M H_2SO_4 solution, respectively. v: 50 mV/s.

Fig. S7 represents E_p vs. log$v$ which is extracted from the CVs recorded at the ranges of 2~500 mV/s for both anodic and cathodic cycles. Both the onset potentials of the anodic and cathodic peaks of S1 shift negatively compared with other electrodes in the acid and alkaline solutions.

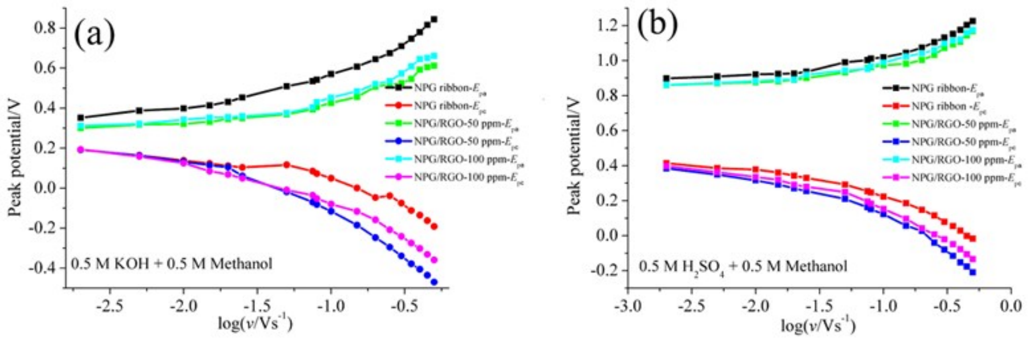

Fig. S7. The diagrams of E_p vs. log$v$, which is extracted from CVs recoded at the ranges of 2-500 mV/s of S0~S2 electrodes in (a) 0.5 M KOH + 0.5 M methanol and (b) 0.5 M H_2SO_4 + 0.5 M methanol solution, respectively.

Fig. S8 shows the CVs of S1 electrode with 50 cycles in alkaline and acid solutions with the scan rate v of 50 mV/s. It can be found that with increasing the cycle, the

current densities of oxidation peaks increase, suggesting that electrode exhibits a good durability. And the current densities in alkaline solution are higher than those in acid solution, indicating that with the electro-oxidation of methanol in alkaline environment is more active than that in acid environment.

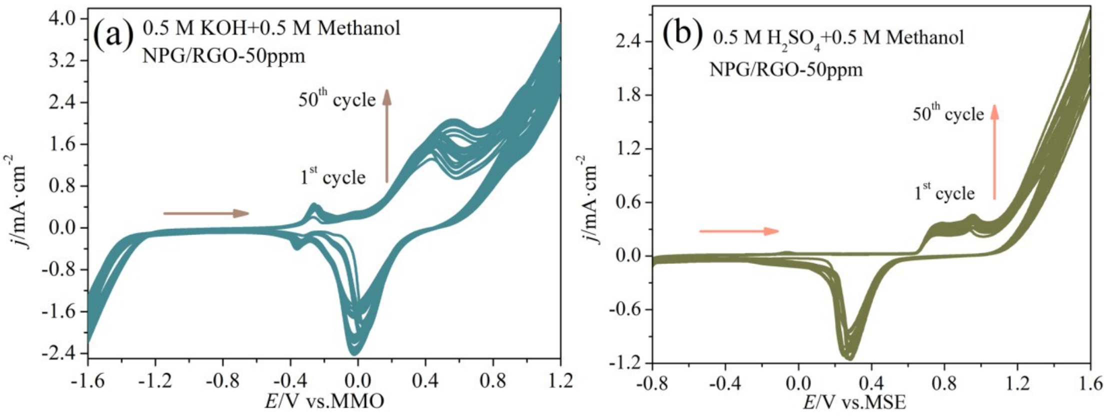

Fig. S8. Cyclic voltammograms (CVs) of S1 electrode (NPG/RGO-50 ppm) with 100 cycles in (a) 0.5 M KOH + 0.5 M CH_3OH solution and (b) 0.5 M H_2SO_4 + 0.5 M methanol solution. Scan rate *v*: 50 mV/s.

The XRD patterns of Al_2Au powders ball-milled from the precursor alloy ingots and standard Au are shown in Fig. S9. It is interesting to find that powders consist of no Al_2Au peaks but only single phase identified as f.c.c. Au-type phase with the broadened (111), (200), (220), and (331) diffractions. We speculate that the constituents of powders contain amorphous phase. The composition of precursor ingot changes from the original Al_2Au phase to the f.c.c. Au-type with broadened diffractions after the high energy ball milling, meaning that certain phase transition happened to precursor ingot in the ball milling process. The same consequence can be seen in the ribbon powders obtained from high energy ball milling, as shown in Fig.

S9 (red line).

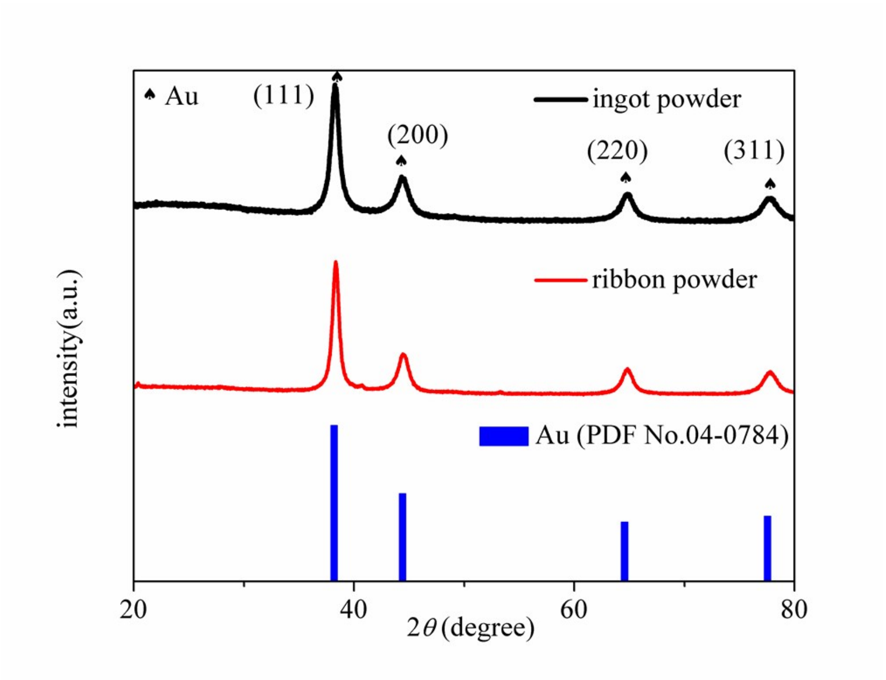

Fig. S9. XRD patterns of the ball-milled precursor ingot and ribbons powders.

XRD results in Fig. S9 demonstrate that the composition of precursor ingot changes from the original Al_2Au phase to the f.c.c. Au-type phase with broadened diffractions after the high energy ball milling. Hence, TEM measurements of precursor ingot powders are carried out in order to further analyze the microstructure. The core-shell structure with black particles as cores and gray cotton fibre-shaped sheets as shells can be seen in Fig. S10a~b. The microstructure of the enlarged cotton fibre-shaped sheets exhibits the dendritic trait (Fig. S10c). The HRTEM image shows that ingot powders contain the amorphous structures, as well as the crystalline grain, and the regular arranged fringes are indicated as f.c.c. Au. Two obvious polycrystalline rings can be seen in the corresponding SAED images, which can be indexed as Al_2O_3 (121), (300), (320), and (311) reflections (PDF No. 52-0803), respectively. In addition, the

diffraction spots in Fig. S10f can be identified as Au_4Al phase (PDF No. 39-0777). The TEM results demonstrate that ingot powders comprise multiple phases, including Al_2O_3, Au, and Au_4Al, etc.

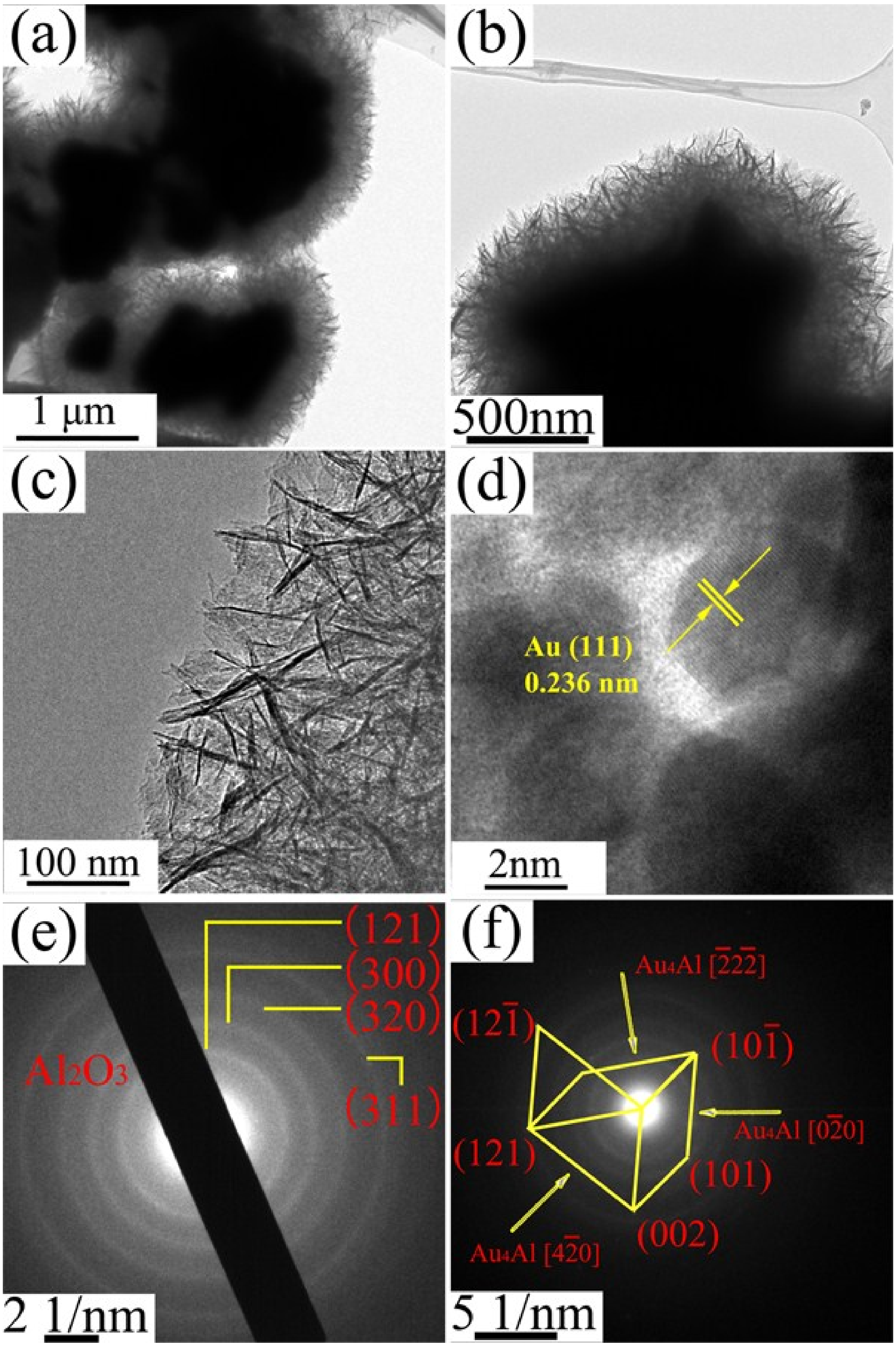

Fig. S10. TEM((a)-(c)), HRTEM(d) and SAED((e)-(f)) images of the precursor

ingot powders.

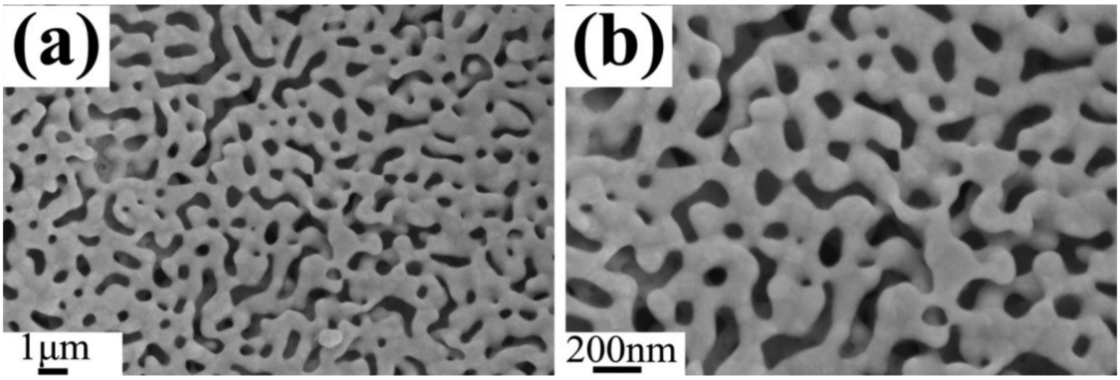

Fig. S11. Plain-view SEM images of P0 powder sample.

Fig. S12 presents SEM images of NPG/RGO nanocomposite powder sample P1 electrode after a series of electrochemical tests. Compared with SEM images of pristine powder sample P1 (Fig. 12), the structure of P1 remains intact after tests and RGO layers can still be attached on NPG surface, implying the marvelous structural stability of NPG/RGO nanocomposite electrodes.

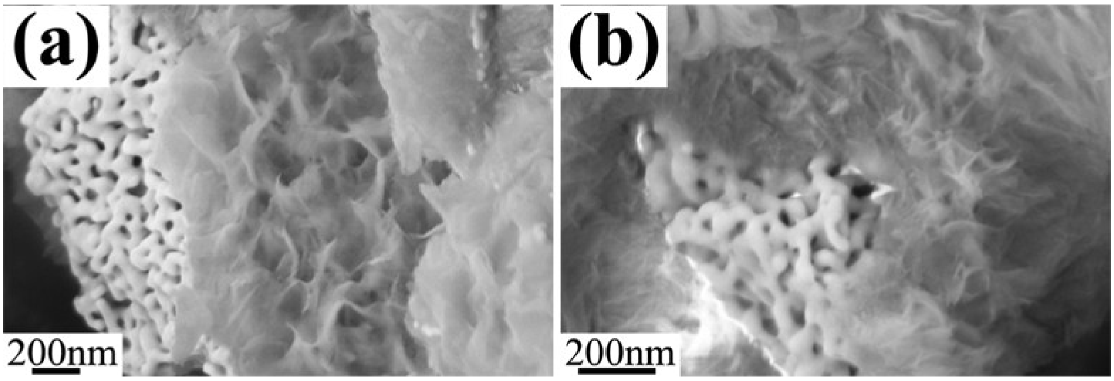

Fig. S12. SEM images of P1 powder sample after electrochemical tests.
